# Clinical and behavioral predictors of HIV-associated neurocognitive disorder among people with HIV who use substances: a secondary analysis of the HOPE trial

**DOI:** 10.1007/s13365-026-01315-9

**Published:** 2026-05-07

**Authors:** Gloria Wang, Xueying Yang, Kesheng Wang, Xiaoming Li, Zheng Dai, Shan Qiao

**Affiliations:** 1 Department of Psychology, College of Arts and Sciences, Emory University, Atlanta, GA 30322 USA; 2 South Carolina SmartState Center for Healthcare Quality, Arnold School of Public Health, University of South Carolina, Columbia, Columbia, SC 29208 USA; 3 Department of Health Promotion, Education, and Behavior, Arnold School of Public Health, University of South Carolina, Columbia, SC 29208 USA; 4 Department of Biobehavioral Health & Nursing Science, College of Nursing, University of South Carolina, 1601 Greene Street, Columbia, SC 29208 USA; 5 Health Affairs Institute, Health Sciences Center, West Virginia University, Morgantown, WV 26506 USA

**Keywords:** HIV-associated neurocognitive disorder (HAND), International HIV Dementia Scale (IHDS), Substance use, Hematologic biomarkers, Cognitive function, Longitudinal analysis

## Abstract

This study examined behavioral, clinical, and hematologic factors associated with HIV-associated neurocognitive disorder (HAND) among people with HIV (PWH) using the International HIV Dementia Scale (IHDS) within a randomized clinical trial. This secondary analysis used data from the Hospital Visit as Opportunity for Prevention and Engagement for HIV-Infected Drug Users (HOPE) study, which enrolled 801 PWH who use substances from 11 U.S. hospitals. CD4 cell count, HIV-1 viral load, Global Severity Index (GSI), Global Assessment of Functioning (GAF), and Physical and Mental Component Scores (PCS, MCS) were assessed at baseline, 6 months, and 12 months. HAND was defined as an IHDS score ≤ 10. Multivariable linear and logistic regression models were used to identify baseline correlates of IHDS score and HAND, respectively. Linear mixed models were applied to evaluate the longitudinal changes in clinical outcomes. HAND prevalence was 76.3% (84.2% for females and 72.5% for males). Multivariable linear models revealed that obesity, CD4 cell counts < 200 cells/ µL, higher hemoglobin level and hematocrit, and higher GAF scores were significantly associated with IHDS score (p < 0.05). Furthermore, logistic models showed that recent alcohol use, obesity, viral suppression (≤ 200 copies/mL), hemoglobin and hematocrit levels, and GAF scores were associated with lower odds of HAND, whereas platelet count was linked to higher odds. Longitudinal analyses demonstrated significant increases in hemoglobin, hematocrit, PCS, and MCS over time, alongside decreases in platelet count and GSI. Compared with participants without HAND, those with HAND consistently exhibited lower hemoglobin, hematocrit, GAF, and GSI scores, and higher platelet counts and MCS scores across follow-up. These findings underscore the complex interplay between hematologic and mental health, substance use, and functional status in shaping neurocognitive outcomes among PWH. Targeting modifiable hematologic, behavioral, and psychosocial factors may help reduce HAND risk and improve long-term cognitive and functional outcomes. ClinicalTrials.gov ID: NCT01612169.

## Introduction

Despite the transformative effect of combination antiretroviral therapy (ART) on the survival and general health of people with HIV (PWH), neurocognitive complications remain a persistent and significant concern. HIV-associated neurocognitive disorder (HAND) continues to be a prevalent comorbidity among PWH, with substantial consequences for ART adherence, everyday functioning, employment, driving performance, and health-related quality of life (Saloner and Cysique 2017). Globally, meta-analyses and systematic reviews reflect wide variability in HAND prevalence estimates typically in the range of ~ 30–50%, but spanning widely from ~ 7% to ~ 88% across different settings, underscoring heterogeneity in methodology, diagnostic criteria, and population characteristics (Ellis et al. 2023; Mekuriaw et al. 2023; Wei et al. 2020).

The concept of HAND encompasses a spectrum of neurocognitive impairment among PWH, ranging from asymptomatic neurocognitive impairment (ANI) to mild neurocognitive disorder (MND) and HIV-associated dementia (HAD), as defined by the Frascati criteria (Antinori et al. 2007). Although HAD has become uncommon in the ART era, milder forms (ANI and MND) remain prevalent and may impact functional capacity (Ellis et al. 2023; Fatokun et al. 2025). Estimates of HAND prevalence varies widely. For example, one meta-analysis of Frascati-based studies reported a pooled prevalence of 44.9% (Wei et al. 2020), while other reviews have reported similarly broad ranges across global and regional samples (Mekuriaw et al. 2023; Zenebe et al. 2022). This variability reflects differences in population characteristics (e.g., age, education, comorbidity burden), HIV-related clinical factors (e.g., nadir CD4, ART adherence), and methodological factors, including the diagnostic instruments and thresholds used (Nightingale et al. 2023; Ellis et al. 2023).

The risk architecture of HAND is multifactorial, encompassing HIV-specific, demographic, psychiatric, vascular, metabolic, and behavioral domains. Among the most consistently identified predictors is nadir CD4 + T-cell count, a marker of historical immunosuppression that reflects cumulative central nervous system (CNS) injury prior to initiation of ART. Ronald J. Ellis et al. (2011) demonstrated that higher nadir CD4 counts were associated with substantially lower odds of neuropsychological impairment, underscoring the critical importance of early ART initiation (Ellis et al. 2011). More recent work by Ronald J. Ellis et al. (2023) further highlights the mechanistic complexity of HAND, emphasizing the roles of persistent immune activation, residual viral replication, and co-occurring metabolic and vascular comorbidities in driving neurocognitive decline despite virologic suppression (Ellis et al. 2023). Demographic and psychosocial factors also contribute substantially to HAND risk. Aging-related processes, including neurodegeneration, increased cerebrovascular burden, and reduced neural plasticity, interact with HIV-associated neuropathology to exacerbate cognitive vulnerability in older PWH (Saloner and Cysique 2017). In addition, psychiatric comorbidities, particularly major depressive disorder and anxiety disorders, are highly prevalent among PWH and are consistently associated with poorer cognitive outcomes. These associations are thought to be mediated through interconnected inflammatory, neuroendocrine, and behavioral pathways (Fatokun et al. 2025; Salis et al. 2024).

Cardiometabolic and vascular disease represent a second major axis of risk for HAND in the contemporary ART era. Conditions such as hypertension, type 2 diabetes, metabolic syndrome, and obesity are increasingly prevalent with aging among PWH and are independently associated with mechanisms that plausibly accelerate neurocognitive decline, including white matter abnormalities, small-vessel cerebrovascular injury, and impaired neurovascular coupling (Ellis et al. 2023; Yu et al. 2019). Evidence from meta-analyses and longitudinal cohort studies indicates that vascular risk factors are linked to substantially higher odds of HAND, as well as poorer performance across executive function, processing speed, and motor domains (McIntosh et al. 2021; Yu et al. 2019). Behavioral factors, particularly substance use, represent a critical and modifiable domain within this risk architecture. Stimulant use (e.g., cocaine) has been shown to potentiate neuroinflammation and front striatal injury, with several cohort studies suggesting additive or synergistic effects with HIV on neurocognitive impairment, although findings remain heterogeneous and are often influenced by co-occurring psychiatric conditions (Kennedy and Zerbo 2014; Meade et al. 2015). Similarly, alcohol use among virally suppressed individuals has demonstrated inconsistent associations with neurocognitive outcomes, likely reflecting variability in dose, patterns of consumption, timing, and comorbidity profiles (Sanmartí et al. 2021). These findings support the inclusion of substance use variables as key behavioral exposures in HAND research.

Psychosocial and functional factors are likewise integral to understanding HAND. Psychiatric symptoms and psychological distress are highly prevalent among PWH and are consistently linked to worse cognitive outcomes through interconnected inflammatory, neuroendocrine, and behavioral pathways (Nightingale et al. 2023; Salis et al. 2024). Moreover, functional measures, such as the Global Assessment of Functioning (GAF) and health-related quality-of-life indices such as physical component scores (PCS) and mental component scores (MCS), capture the real-world impact of cognitive impairment and align with established HAND frameworks that emphasize both neurocognitive performance and functional decline (Saloner and Cysique 2017). Additionally, the Global Severity Index (GSI) provides an index of psychiatric distress that is frequently incorporated into HAND-related research (Rytilä-Manninen et al. 2016). Accordingly, inclusion of these measures enables a more comprehensive assessment of both determinants and consequences of HAND.

Emerging evidence also highlights hematologic indices as accessible systemic biomarkers linked to HAND. A landmark study by Sanjay Kallianpur et al. (2016) demonstrated that anemia was associated with incident HAND, while elevated mean corpuscular volume (MCV) and mean corpuscular hemoglobin (MCH) were correlated with poorer cognitive performance, even after adjustment for potential confounders (Kallianpur et al. 2016). These associations may reflect underlying mechanisms such as chronic hypoxia, iron dysregulation, mitochondrial dysfunction, and systemic inflammation (Ellis et al. 2023; Kallianpur et al. 2016).

Given the high prevalence and burden of HAND, effective screening for neurocognitive impairment and its functional impact is critical. The International HIV Dementia Scale (IHDS) is a brief, cross-cultural bedside screening tool that has demonstrated feasibility and utility across diverse settings, including its original validation in the U.S. and Uganda (Sacktor et al. 2005). Owing to its brevity and ease of administration, the IHDS is widely used to identify individuals at risk for HAND, particularly in clinical and research contexts where comprehensive neuropsychological testing is not feasible. A raw IHDS cut-point of ≤ 10 is commonly applied as a standard screening threshold (Goodkin et al. 2014; Marin-Webb et al. 2016; Milanini et al. 2018; Nascimento et al. 2025; Riedel et al. 2006; Rosca et al. 2021) although subsequent studies have reported variability in sensitivity and specificity depending on the population and cut-point used (Haddow et al. 2013; Marin-Webb et al. 2016; Milanini et al. 2018; Rosca et al. 2021). For example, a cut-off of ≤ 11 may improve sensitivity, whereas ≤ 10 may increase specificity (Marin-Webb et al. 2016). While the IHDS does not provide a definitive diagnosis and may be less sensitive for milder forms of impairment, it remains a practical and valuable screening instrument for identifying individuals who may benefit from further evaluations.

Despite substantial progress, critical gaps remain. Prevalence of HAND estimates vary widely across tools and contexts; the contribution of modifiable systemic factors particularly hematologic and cardiometabolic markers remains under-characterized; and the cognitive effects of using substances such as alcohol and stimulants are insufficiently understood in virally suppressed populations (Meade et al. 2015). Few adequately powered longitudinal or interventional studies integrate multi-domain outcomes spanning cognition, functioning, mental health, biomarkers. Moreover, evolving ART landscapes and earlier treatment initiation necessitate updated frameworks to disentangle HIV-specific, vascular, aging-related, and cognitive-reserve mechanisms (Ellis et al. 2023; McIntosh et al. 2021; Nightingale et al. 2023). The present study aimed to examine how HAND relates to routine hematologic markers including hemoglobin, hematocrit, and platelet count, substance use, psychosocial, and functional measures in a randomized clinical trial.

## Methods

### Data source

This study is a secondary data analysis from the Hospital Visit as Opportunity for Prevention and Engagement for HIV-Infected Drug Users (HOPE) randomized clinical trial conducted in the National Institute on Drug Abuse (NIDA) Clinical Trials Network (CTN-0049), which evaluated the effectiveness of a brief intervention to improve HIV care engagement among PWH who use substances across 11 hospitals in the United States (Metsch et al. 2016). The HOPE trial enrolled 801 PWH who use substances from 11 U.S. hospitals between 2012 and 2017. Participants must meet all the following criteria: (1) Be hospitalized and live with HIV at the time of recruitment. (2) Be 18 years of age or older. (3) Meet at least one of the following clinical conditions: (a) Have an AIDS-defining illness during the current hospital admission; (b) Have a CD4 count < 350 cells/µL and HIV viral load > 200 copies/mL within the past 6 months; or (c) Have a CD4 count ≤ 500 cells/µL and viral load > 200 copies/mL (or unknown) within the past 12 months, with the Site Principal Investigator (PI) determining that the patient likely has a current viral load > 200 copies/mL, is not effectively taking antiretroviral therapy (ART), and requires ART. (4). Report or have documented opioid use, stimulant use, or heavy alcohol use within the past 12 months. (5) Have a Karnofsky Performance Status score ≥ 60. (6). Be able to provide informed consent, complete baseline procedures, provide locator information and HIPAA authorization, reside locally for follow-up, and communicate in English. Individuals will be excluded if they: (1) Do not meet the inclusion criteria. (2) Are unable to provide informed consent due to cognitive impairment. (3) Are withdrawn at the discretion of the Site PI, with approval from the Lead Investigator. For this secondary analysis, we included participants with complete baseline data on the IHDS, hematologic variables, and psychosocial measures. Participants were assessed at baseline, 6 months, and 12 months, with standardized clinical, psychosocial, and laboratory evaluations. All participants provided written informed consent under Institutional Review Board (IRB) approval at each participating institution, consistent with ethical principles of the Declaration of Helsinki. The current study was exempted from IRB review due to secondary data analysis using publicly available databases.

### HIV-associated neurocognitive disorder (HAND) screening

Cognitive function was assessed using the IHDS, a validated three-item instrument measuring motor speed (0–4 points), psychomotor speed (0–4 points), and memory recall (0–4 points) (Sacktor et al. 2005). The total IHDS scores range from 0 to 12, with values ≤ 10 indicating probable HAND (Marin-Webb et al. 2016; Rosca et al. 2021). The IHDS has shown good sensitivity and specificity for detecting subcortical cognitive impairment among PWH across diverse settings, including low- and middle-income countries (Haddow et al. 2013; Nightingale et al. 2023).

### Demographic information

Participants provided demographic information through a self-reported questionnaire. Age was categorized into four groups (18–25 years, 26–49 years, 50–60 years and 61 years or older). Sex was classified as male or female. Race/ethnicity included three groups (Hispanic, non-Hispanic African American, and non-Hispanic White and other). Marital status was categorized as never married, widowed/divorced/separated, and married or cohabiting. Educational level was recorded as ≤ high school or ≥ high school, and employment was recorded as currently working (Yes) and unemployed (No).

### Clinical and laboratory variables

Clinical data included plasma HIV-1 RNA levels (copies/mL) and CD4 cell counts, extracted from medical records. CD4 cell count and viral load were measured using standard clinical assays performed in CLIA-certified laboratories. CD4 cell count and HIV-1 viral load were assessed at baseline, 6 months, and 12 months. HIV viral suppression was defined as having a viral load of ≤ 200 copies/mL. CD4 cell count was categorized as a binary variable (< 200 cells/ µL and ≥ 200 cells/ µL). Hemoglobin, hematocrit, and platelet counts were obtained as part of routine complete blood count testing using automated hematology analyzers at each site. Laboratory biomarkers were selected based on evidence linking hematologic indices to neurocognitive performance in HIV (Kallianpur et al. 2016; Okwuegbuna et al. 2023; Ragin et al. 2011). This study included hemoglobin (g/dL), hematocrit (%), and platelet count (×10⁹/L), all measured using standard hospital laboratory assays. Lower hemoglobin and platelet levels have been associated with greater cognitive impairment in PWH, supporting their inclusion as potential predictors. Clinical labs data were measured at baseline, 6 and 12 months. Body mass index (BMI) was calculated as weight (kg) divided by square of height (m²), with obesity defined as BMI ≥ 30 kg/m².

## Psychosocial and functional measures

Psychosocial and functional measures were included given their established associations with neurocognitive impairment and HAND among PWH, and providing a more comprehensive assessment of factors potentially related to neurocognitive outcomes.

### Global severity index (GSI)

Psychological distress was measured using the GSI from the Brief Symptom Inventory (BSI-18), which is a brief, easy-to-administer, abbreviated version of the BSI (Derogatis 2011; Recklitis et al. 2006). The BSI-18 consists of 18 five-point scale items (0 = “not at all,” to 4 = “extremely”) evaluating the degree of distress experienced over the past seven days. The sum of responses yields a GSI. The GSI is a continuous score reflecting overall psychological symptom burden, with higher values indicating greater distress. The GSI was measured at baseline, 6 months, and 12 months.

### Global assessment of functioning (GAF)

Functional status was evaluated with the GAF scale, a clinician-rated measure ranging from 0 to 100 that captures psychological, social, and occupational functioning (Aas 2010; Yamauchi et al. 2001). Higher GAF scores indicate better overall functioning. GAF assessments were conducted at baseline, 6 months, and 12 months.

### Perceived health

Both perceived physical and mental health were assessed using the SF-12 health survey, a validated 12-item instrument derived from the SF-36. Items cover domains such as physical functioning, physical role, bodily pain, general health, vitality, social functioning, role emotional, and mental health (Ware et al. 1996). Responses are used to calculate PCS and MCS, with higher scores reflecting better perceived health. SF-12 was administered at baseline, 6 months, and 12 months.

### Substance use and health behaviors

Substance use variables were included due to their known relevance to neurocognitive outcomes in PWH, as substance use may independently affect cognition and interact with HIV-related factors. Substance use was self-reported using a modified version of the Timeline Follow-Back method (Sobell and Sobell 1992), capturing frequency and type of substances used within the past 30 days, including alcohol, heroin, cocaine, marijuana, and other illicit drugs. Substance use in the past year was defined by this question: “How many times in the past year have you used an illegal drug or used a prescription medication for nonmedical reasons?” Responses were coded as binary (Yes/No) for specific substances including cocaine, heroin, rock or crack cocaine, and marijuana. Current cigarette smoking was also recorded as a binary variable (yes/no).

### Statistical analysis

Categorical variables were presented as frequencies and percentages, while continuous variables were presented as mean and standard deviation (SD). Baseline differences between participants with and without probable HAND (IHDS ≤ 10) were assessed using independent t-tests for continuous variables, and χ² tests for categorical variables. Multivariable linear regression models were used to identify baseline correlates of IHDS score as a continuous outcome. Multivariable logistic regression (MLR) models with adjusted odd ratio (aOR) and their 95% confidence intervals (95% CI) were used to identify baseline predictors of HAND status (IHDS ≤ 10 vs. > 10). Predictors included age, sex, race/ethnicity, education, substance use (alcohol, cocaine, heroin, marijuana, smoking), obesity, CD4 cell count, hemoglobin, hematocrit, platelet count, GSI, GAF, PCS, and MCS (Kallianpur et al. 2016; Okwuegbuna et al. 2023). Longitudinal changes in clinical and psychosocial measures across the three time points (baseline, 6 months, 12 months) were assessed using linear mixed model, with time as a within-subject factor and HAND status as a between-subject factor. Interaction terms tested whether changes over time differed by HAND group. All models were adjusted for key demographic covariates (age, sex, education, race/ethnicity). R packages MASS (Venables and Ripley 2002) and SPSS Statistics for Windows, Version 29.0 (IBM Corp., Armonk, NY) were used for data analysis. All statistical tests were two-tailed, with *p* < 0.05 considered statistically significant.

## Results

### Participant characteristics

Table 1 shows the descriptive statistics (mean, SD and range) of IHDS and 3 components. As shown in Table 2, the overall prevalence of HAND among PWH was 76.3%, with a notably higher rate among females (84.2%) compared with males (72.5%). A clear age-related gradient was observed, with prevalence increasing across age categories: 52.5% among individuals aged 18–25, 78.0% in those aged 26–49, 76.3% among participants aged 50–60, and 81.3% among those aged 61 years and older. Higher prevalence was also observed among participants with lower educational attainment (81.4%), those who were unemployed (77.7%), and individuals identifying as non-Hispanic African American (80.7%).

**Table 1:** Descriptive statistics of IHDS and 3 components

Variable	Total (*N*)	Mean ± SD	Range
Motor speed	801	2.38 ± 1.16	4
Psychomotor speed	799	2.86 ± 1.14	4
Memory recall	801	3.56 ± 0.82	4
IHDS	799	8.80 ± 2.19	11

*IHDS* International HIV dementia scale,* SD* Standard deviation

**Table 2:** Prevalence of HAND across demographics

Variable	Total (*N*)	Non-HAND	HAND	Prevalence (%)	*p*-value
Sex					
Female	260	41	219	84.2	< 0.0001
Male	539	148	391	72.5	
Age group (years)					
18–25	40	19	21	52.5	0.0033
26–49	495	109	386	78.0	
50–60	232	55	177	76.3	
61+	32	6	26	81.3	
Race /ethnicity					
Hispanic	88	34	54	61.4	< 0.0001
Non-Hispanic African American	597	115	482	80.7	
Non-Hispanic Whites + Other	114	40	74	64.7	
Education					
< High School	318	59	259	81.4	0.0058
≥ High School	481	130	351	73.0	
Marital status					
Never married	528	123	405	76.7	0.0939
Widowed/Divorced/Separated	181	37	144	79.6	
Married or cohabiting	90	29	61	68.7	
Employment					
No	704	157	547	77.7	0.0099
Yes	93	32	61	65.6	
Overall	799	189	610	76.3	

*HAND* HIV-associated neurocognitive disorder, p**-**value is based on χ^2^ test

Substance use patterns showed differential associations with HAND (Table 3). Baseline use of rock or crack cocaine during the past year was associated with a higher prevalence of HAND (79.1%). Individuals with viral suppression had lower HAND prevalence (66.4%). Moreover, independent samples *t*-tests indicated significant differences in key laboratory and functional measures between groups. Individuals with HAND had statistically significantly lower hemoglobin levels, lower hematocrit, and lower GAF scores, but higher platelet counts compared with those without HAND (*p* < 0.05).

**Table 3:** Descriptive statistics by substance use and clinical factors

Variable	Total (*N*)	Non-HAND (*N*/mean ± SD)	HAND (*N*/mean ± SD)	Prevalence (%)/t-value	*p*-value
Alcohol use in the past 30 days					
No	338	72	266	78.7	0.1748
Yes	460	117	343	74.6	
Cocaine use in the past 30 days					
No	499	123	376	75.4	0.4364
Yes	297	66	231	77.7	
Heroin use in the past 30 days					
No	698	160	538	77.1	0.1812
Yes	100	29	71	71.0	
Marijuana use in the past 30 days					
No	555	127	428	77.1	0.3864
Yes	241	62	179	74.3	
Current smoking					
No	236	52	184	78.0	0.4852
Yes	563	137	426	75.7	
Cocaine use in the last year					
No	416	96	320	76.9	0.2349
Yes	231	83	168	75.4	
Heroin use in the last year					
No	511	118	393	76.9	0.0894
Yes	136	41	95	69.9	
Rock or Crack Cocaine in last year					
No	298	86	212	71.1	0.0193
Yes	349	73	276	79.1	
Marijuana use in last year					
No	292	77	215	73.6	0.3362
Yes	355	82	273	76.9	
Obesity					
Normal	709	162	547	77.2	0.0787
Obesity	86	27	59	68.5	
CD4 cell count < 200 cells/µl					
No	271	69	202	74.5	0.3893
Yes	528	120	408	77.3	
Viral suppression ≤ 200 copies/mL					
No	712	160	552	77.5	0.0244
Yes	87	29	58	66.4	
Hepatitis C virus positive					
No	528	117	411	77.8	0.3436
Yes	258	65	193	74.8	
Hemoglobin (mean ± SD)	738	11.69 ± 1.87	11.18 ± 2.11	3.05	0.0024
Hematocrit (mean ± SD)	738	35.42 ± 5.47	34.28 ± 6.17	2.67	0.0078
Platelets (mean ± SD)	738	209.48 ± 112.01	233.17 ± 117.49	-2.42	0.0156
GAF (mean ± SD)	796	83.37 ± 10.91	77.29 ± 10.93	6.68	< 0.0001
PCS (mean ± SD)	796	36.54 ± 11.94	35.15 ± 10.76	1.69	0.0922
MCS (mean ± SD)	796	41.12 ± 13.75	42.94 ± 12.78	-1.62	0.1066
GSI (mean ± SD)	796	24.15 ± 17.05	21.97 ± 15.80	1.62	0.1057

*HAND* HIV-associated neurocognitive disorder, *GSI* Global severity index; *GAF* Global assessment of functioning; *PCS* Physical component scores;* MCS* Mental component scores; *SD* Standard deviation; p**-**value is based on χ^2^ test/t-test

### The relationship between potential risk factors and IHDS

Table 4 summarizes the results of multivariable linear regression analyses examining factors associated with overall IHDS scores and its three component domains. In domain-specific analyses, marijuana use in the past year, obesity, CD4 cell count < 200 cells/µL, hepatitis C status, and GAF scores were significantly associated with the motor speed component. Psychomotor speed was significantly associated with viral suppression status, hemoglobin and hematocrit levels, as well as GAF and MCS scores (all *p* < 0.05). In contrast, no variables were significantly associated with the memory recall component. For the overall IHDS score, multivariable models indicated that obesity, CD4 cell count < 200 cells/µL, higher hemoglobin and hematocrit levels, and higher GAF scores were independently associated with IHDS performance (all *p* < 0.05).

**Table 4:** Multiple linear regression analyses of factors with IHDS and 3 components

Variable	Motor speed (β ± SE)	Psychomotor speed (β ± SE)	Memory recall (β ± SE)	IHDS (β ± SE)
Alcohol use in the past 30 days(ref = No)	0.05 ± 0.08	0.07 ± 0.08	-0.02 ± 0.06	0.10 ± 0.15
Cocaine use in the past 30 days(ref = No)	0.04 ± 0.09	0.05 ± 0.09	0.01 ± 0.06	0.08 ± 0.16
Heroin use in the past 30 days(ref = No)	0.23 ± 0.12	0.20 ± 0.12	0.01 ± 0.09	0.42 ± 0.23
Marijuana use in the past 30 days(ref = No)	-0.02 ± 0.09	0.12 ± 0.09	0.02 ± 0.06	0.11 ± 0.17
Current smoking (ref = No)	0.09 ± 0.09	0.13 ± 0.097	0.04 ± 0.06	0.25 ± 0.17
Heroin use in the past year (ref = No)	0.201 ± 0.11	0.12 ± 0.11	-0.02 ± 0.03	0.31 ± 0.21
Cocaine use in the past year (ref = No)	0.02 ± 0.10	0.04 ± 0.09	0.01 ± 0.06	0.05 ± 0.17
Rock or Crack Cocaine in last year (ref = No)	-0.01 ± 0.10	0.12 ± 0.10	0.02 ± 0.06	0.15 ± 0.17
Marijuana use in the past year(ref = No)	0.19 ± 0.10*	0.01 ± 0.01	-0.01 ± 0.06	-0.19 ± 0.17
Obesity (ref = No)	0.25 ± 0.13*	0.18 ± 0.13	0.09 ± 0.10	0.52 ± 0.25*
CD4 cell count < 200 cells/µL (ref = No)	-0.26 ± 0.09**	-0.12 ± 0.09	-0.07 ± 0.06	-0.44 ± 0.16**
Viral suppression ≤ 200 copies/mL (ref = No)	0.419 ± 0.13	0.26 ± 0.13*	0.01 ± 0.09	0.45 ± 0.24
Hepatitis C virus positive(ref = No)	0.20 ± 0.10*	0.04 ± 0.09	0.05 ± 0.07	0.21 ± 0.17
Hemoglobin	0.02 ± 0.02	0.05 ± 0.02*	0.02 ± 0.01	0.09 ± 0.04*
Hematocrit	0.01 ± 0.01	0.02 ± 0.01**	0.01 ± 0.01	0.03 ± 0.01*
Platelets	-0.01 ± 0.01	-0.01 ± 0.01	-0.01 ± 0.01	0.01 ± 0.01
GAF	0.02 ± 0.01***	0.02 ± 0.01***	0.01 ± 0.01	0.05 ± 0.01***
PCS	0.01 ± 0.01	0.01 ± 0.01	0.01 ± 0.01	0.01 ± 0.01
MCS	-0.01 ± 0.01	-0.01 ± 0.01*	-0.01 ± 0.01	-0.01 ± 0.01
GSI	0.01 ± 0.01	0.01 ± 0.01	0.01 ± 0.01	0.01 ± 0.01

*IHDS* International HIV dementia scale,* GSI* Global severity index; *GAF* Global assessment of functioning; *PCS* Physical component scores; *MCS* Mental component scores; β adjusted regression coefficient; *SE* Standard error; *OR* Odds ratio; *CI* confidence interval; p**-**value is based on logistic regression

* *p* < 0.05; ** *p* < 0.01, *** *p* < 0.001

### The relationship between potential risk factors and HAND

Table 5 presents the logistic regression results for factors associated with HAND. In the bivariate logistic model, rock or crack cocaine use in the past year and higher platelet counts were associated with increased odds of HAND. In contrast, viral suppression status, higher hemoglobin and hematocrit levels, and higher GAF scores were associated with reduced odds of HAND.

**Table 5:** Univariate and multiple logistic regression analyses of factors associated with HAND

Variable	(β ± SE)	Crude OR (95% CI)	(β ± SE)	Adjusted OR (95% CI)
Alcohol use in the past 30 days(ref = No)				
Yes	-0.23 ± 0.13	0.79(0.57–1.02)	-0.36 ± 0.18	0.70(0.49–0.99)*
Cocaine use in the past 30 days(ref = No)				
Yes	0.14 ± 0.17	1.15(0.81–1.61)	-0.07 ± 0.17	0.93(0.65–1.33)
Heroin use in the past 30 days(ref = No)				
Yes	-0.32 ± 0.24	0.73(0.46–1.16)	-0.43 ± 0.44	0.65(0.40–1.09)
Marijuana use in the past 30 days(ref = No)				
Yes	-0.15 ± 0.17	0.86(0.60–1.22)	-0.16 ± 0.19	0.86(0.59–1.24)
Current smoking (ref = No)				
Yes	-0.13 ± 0.19	0.88(0.61–1.26)	-0.26 ± 0.20	0.77(0.53–1.13)
Heroin use in the past year (ref = No)				
Yes	-0.36 ± 0.21	0.70(0.46–1.06)	-0.44 ± 0.23	0.64(0.41–1.01)
Cocaine use in the past year (ref = No)				
Yes	0.22 ± 0.19	0.80(0.55–1.15)	-0.13 ± 0.20	0.88(0.60–1.29)
Rock or Crack Cocaine in last year (ref = No)				
Yes	0.43 ± 0.18	1.53(1.07–2.20)*	0.10 ± 0.20	1.10(0.75–1.63)
Marijuana use in the past year(ref = No)				
Yes	0.18 ± 0.18	1.198(0.83–1.71)	-0.23 ± 0.20	126(0.85–1.87)
Obesity (ref = No)				
Yes	-0.44 ± 0.25	0.64(0.40–1.05)	-0.67 ± 0.27	0.51(0.30–0.86)*
CD4 cell count < 200 cells/µl (ref = No)				
Yes	0.15 ± 0.17	1.26(0.83–1.63)	0.30 ± 0.18	1.35(0.94–1.93)
Viral suppression ≤ 200 copies/mL (ref = No)				
Yes	-015 ± 0.25	0.58(0.40–0.94)*	-0.67 ± 0.26	0.51(0.31–0.86)**
Hepatitis C virus positive(ref = No)				
Yes	-0.16 ± 0.18	0.85(0.60–1.20)	-0.18 ± 0.19	0.83(0.57–1.22)
Hemoglobin	-0.14 ± 0.04	0.88(0.83–0.96)**	-0.08 ± 0.04	0.92(0.84–0.99)*
Hematocrit	-0.05 ± 0.01	0.96(0.94–0.98)***	-0.03 ± 0.21	0.97(0.95–1.05)
Platelets	0.01 ± 0.01	1.00(1.00-1.01)*	0.01 ± 0.01	1.01(1.00-1.01)*
GAF	-0.04 ± 0.01	0.95(0.94–0.97)***	-0.04 ± 0.01	0.96(0.94–0.97)***
PCS	-0.01 ± 0.01	0.99(0.98-1.00)	-0.01 ± 0.01	0.99(0.98–1.01)
MCS	0.01 ± 0.01	1.01(0.99–1.02)	0.02 ± 0.01	1.02(0.99–1.03)
GSI	-0.01 ± 0.01	0.99(0.99-1.00)	-0.01 ± 0.01	0.99(0.98–1.01)

*HAND* HIV-associated neurocognitive disorder, *GSI* Global severity index; *GAF* Global assessment of functioning; *PCS* Physical component scores; *MCS* Mental component scores; *OR* Odds ratio; *CI* confidence interval; p**-**value is based on logistic regression

After adjusting for demographic characteristics, the multivariable logistic regression model identified several protective behavioral and clinical factors. Alcohol use in the past 30 days (OR = 0.70, 95% CI [0.49, 0.99]), obesity (OR = 0.50, 95% CI [0.30, 0.86]), viral suppression status (OR = 0.51, 95% CI [0.31, 0.86]), higher hemoglobin levels (OR = 0.92, 95% CI [0.84, 0.99]), and higher GAF scores (OR = 0.96, 95% CI [0.94, 0.97]) were also inversely associated with HAND. Conversely, higher platelet counts (OR = 1.01, 95% CI [1.00, 1.01]) remained significantly associated with increased odds of HAND (*p* < 0.05).

### Longitudinal changes in clinical variables

**Fig. 1:**
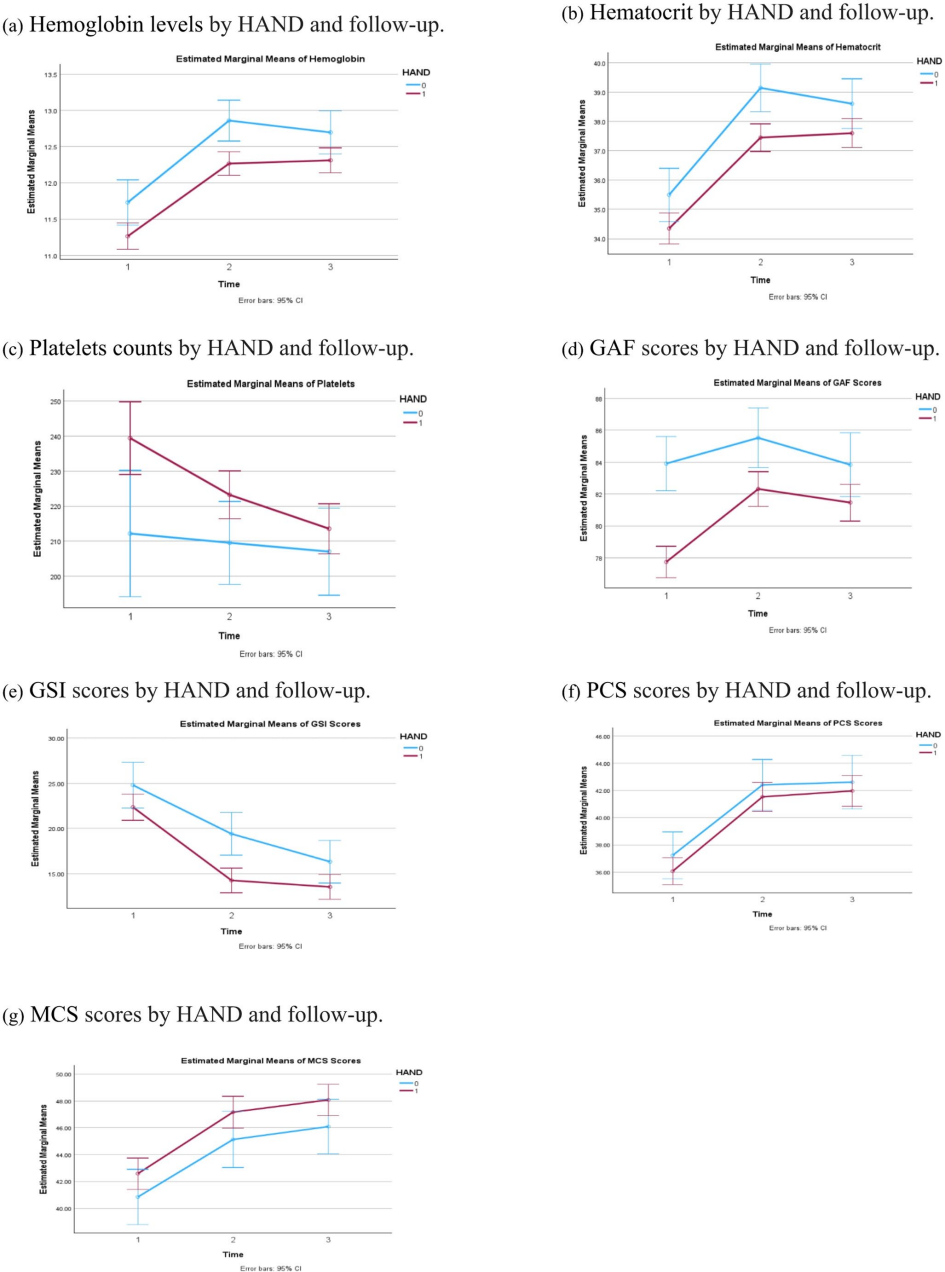
Clinical outcomes at baseline (time 1), 6-month (time 2), and 12-month (time 3) follow-up by HAND status. HAND: HIV-associated neurocognitive disorder. GSI: Global severity index; GAF: Global Assessment of Functioning; PCS: Physical component scores; MCS: Mental component scores

Table 6 summarizes the results of linear mixed models examining longitudinal changes in seven clinical outcomes, and Fig. 1 visualizes these trajectories by HAND status. Longitudinal analyses demonstrated significant increases in hemoglobin, hematocrit, PCS, and MCS over time, alongside decreases in platelet count and GSI. Compared with participants without HAND, those with HAND consistently exhibited lower hemoglobin, hematocrit, GAF, and GSI scores, and higher platelet counts and MCS scores across follow-up.

**Table 6:** Clinical Outcomes at Baseline and Follow-up by HAND Status

Variable		Overall Mean ± SE	Baseline Mean ± SE	6 months Mean ± SE	12 months Mean ± SE	F^1^, *p*-value^1^
Hemoglobin	Non-HAND	12.43 ± 0.13	11.73 ± 0.16	12.86 ± 0.14	12.70 ± 0.15	122.91, < 0.0001
	HAND	11.95 ± 0.07	11.26 ± 0.09	12.27 ± 0.08	12.31 ± 0.09	
	F^2^ / p-value^2^	10.61, < 0.0001	6.48, 0.0112	12.82, < 0.0001	4.83, 0.0282	
Hematocrit	Non-HAND	37.75 ± 0.36	35.50 ± 0.46	39.15 ± 0.42	38.61 ± 0.43	122.15, < 0.0001
	HAND	36.47 ± 0.21	34.35 ± 0.27	37.45 ± 0.24	37.60 ± 0.25	
	F^2^ / p-value^2^	9.33, 0.0023	4.61, 0.0321	12.50, < 0.0001	4.07,0.0442	
Platelets	Non-HAND	209.58 ± 5.80	212.18 ± 9.17	209.552 ± 6.02	207.03 ± 6.82	9.61, 0.0020
	HAND	225.39 ± 3.53	239.39 ± 5.27	223.22 ± 3.46	213.55 ± 3.63	
	F^2^ / p-value^2^	5.59, 0.0184	6.61, 0.0103	3.88, 0.0492	0.80, 0.3709	
GAF	Non-HAND	84.43 ± 0.75	83.91 ± 0.87	85.53 ± 0.95	83.84 ± 1.02	14.67, < 0.0001
	HAND	80.51 ± 0.44	77.74 ± 0.50	82.32 ± 0.55	81.47 ± 0.59	
	F^2^ / p-value^2^	20.19, < 0.0001	37.71, < 0.0001	8.51, 0.0821	4.07, 0.0442	
GSI	Non-HAND	20.18 ± 0.97	24.80 ± 1.29	19.41 ± 1.20	16.33 ± 1.21	112.91, < 0.0001
	HAND	16.72 ± 0.56	22.36 ± 0.74	14.26 ± 0.69	13.53 ± 0.70	
	F^2^ / p-value^2^	9.42, 0.0022	2.68, 0.1022	13.80, < 0.0001	4.02, 0.0455	
PCS	Non-HAND	40.75 ± 0.76	37.28 ± 0.88	42.41 ± 0.95	42.61 ± 1.01	62.64, < 0.0001
	HAND	39.85 ± 0.43	36.06 ± 0.50	41.53 ± 0.54	41.97 ± 0.58	
	F^2^ / p-value^2^	1.05, 0.3057	1.33, 0.2502	0.64, 0.4225	0.31, 0.5800	
MCS	Non-HAND	44.02 ± 0.79	40.85 ± 1.05	45.13 ± 1.06	46.09 ± 1.03	54.81, < 0.0001
	HAND	45.94 ± 0.45	42.58 ± 0.59	47.16 ± 0.60	48.02 ± 0.59	
	F^2^ / p-value^2^	4.44, 0.0355	2.08, 0.1494	2.78, 0.0960	2.81, 0.0944	

*HAND* HIV-associated neurocognitive disorder, *GSI* Global severity index; *GAF* Global assessment of functioning; *PCS* Physical component scores; *MCS* Mental component scores; *SE* Standard error; F^1^ F value based on F test for comparing 3 time points from linear mixed model; p-value^1^ p**-**value for comparing 3 time points based on F test from linear mixed model; F^2^ F value based on F test for comparing HAND with non-HAND from linear mixed model; p-value^2^ p**-**value for comparing HAND with non-HAND based on F test from linear mixed model

## Discussion

Among 801 people living with HIV, the prevalence of HAND was high (76.3%), with a disproportionate burden among females, older adults, individuals with lower educational attainment, the unemployed, and non-Hispanic African Americans. Substance use showed mixed associations; recent crack/cocaine use was linked to higher HAND prevalence, whereas viral suppression was associated with lower prevalence. In adjusted analyses, multiple behavioral, clinical, and hematologic factors were significantly associated with neurocognitive performance. Motor and psychomotor speed were influenced by substance use, CD4 counts < 200 cells/µL, viral suppression, hematologic markers, and functional status, while memory recall showed no significant associations. Multivariable models indicated that alcohol use, obesity, viral suppression, higher hemoglobin levels, and better functioning were associated with reduced odds of HAND, whereas elevated platelet count increased risk. Longitudinally, hematologic and quality-of-life measures improved, but individuals with HAND exhibited persistently poorer clinical and functional outcomes.

### Prevalence and population context

Our observed HAND prevalence (76.3%) is higher than many pooled estimates but consistent with other clinic-based and population-specific reports, which vary widely (~ 20–70%) depending on sample composition, assessment instruments, and regional context (e.g., ART access, CD4). Large meta-analyses estimate a global pooled prevalence of HAND of approximately 42.6% (Wang et al. 2020), and other systematic reviews report substantial heterogeneity across settings (Wei et al. 2020; Zenebe et al. 2022). Clinic-based cohorts that include a higher proportion of older individuals, those with medical or psychiatric comorbidities, lower education attainment, or socioeconomic disadvantage, often report markedly higher HAND prevalence, which helps contextualize our findings (Wang et al. 2020; Zenebe et al. 2022). Furthermore, the higher prevalence among women is consistent with findings from several U.S. and international cohorts (Maki and Martin-Thormeyer 2009; Rubin et al. 2018). Sex differences may be due to differential ART adherence, higher prevalence of comorbidities, and social disadvantage (Maki et al., 2018). Consistent with prior studies, we observed that older age, African American race, lower education, and unemployment were associated with greater risk of HAND. However, the observed HAND prevalence (76.3%) is higher than previous studies due to PWH who use substances. These findings underscore the influence of sociodemographic determinants on neurocognitive health among PWH (Gorman et al. 2009; Petersen et al. 2023).

### Hematologic factors and neurocognitive function

The association of lower hemoglobin and hematocrit with HAND in our cohort aligns with multicenter evidence linking anemia and red-cell indices to neurocognitive decline in the ART era (Kallianpur et al. 2016; Ku et al. 2014). Anemia may contribute through mechanisms plausibly relevant to psychiatric symptomatology, such as reduced cerebral oxygen delivery, iron dysregulation, mitochondrial dysfunction, or by serving as an inflammatory marker coinciding with systemic immune activation (Kallianpur et al. 2016). Although hemoglobin levels differed statistically between groups, the magnitude of this difference was small and remained within the normal clinical range, suggesting limited clinical significance. In addition, anemia was not formally defined or assessed in this study, and thus it is unclear how many participants met diagnostic criteria for anemia versus exhibiting relatively lower, but still normal, hemoglobin levels.

Our finding of higher baseline platelet counts among participants with HAND adds to the existing literature implicating platelet activation, platelet–monocyte complexes, and platelet-mediated endothelial dysfunction in HIV-related neuroinflammation (Mesquita et al. 2018; Singh et al. 2014). Previous studies have found that greater platelet reactivity (without antiplatelet therapy) was associated with a higher risk of late-life dementia and poorer cognitive and functional status (Chiva-Blanch et al. 2021; Ramos-Cejudo et al. 2022). Platelet activation could therefore represent a peripheral biomarker of neurovascular or inflammatory processes contributing to cognitive dysfunction. Although we observed a statistically significant association between higher platelet count and HAND, the direction of this finding differs from some prior studies that have reported decreased platelet counts in this population, though literature remains inconsistent on this issue. Importantly, the magnitude of the difference in platelet count between those with and without HAND in our sample was small and within the normal clinical range, suggesting limited clinical significance. Therefore, these findings should be interpreted cautiously and not as evidence of a clinically meaningful alteration in platelet levels. In addition, while platelet activation has been proposed as a potential mechanism linking hematologic factors to neurocognitive outcomes, the present study did not include direct measures of platelet activation. As such, platelet count should be considered a limited and indirect marker, and no conclusions can be drawn regarding platelet activation or its role in HAND from these data. Future studies incorporating more specific biomarkers of platelet function and activation are needed to clarify these relationships.

### Substance use and cognitive vulnerability

The relationships between substance use and cognition were heterogeneous. Cocaine use was associated with increased odds of HAND in our bivariate analyses, consistent with studies showing that cocaine exacerbates HIV-related deficits in verbal memory and learning (Litvin et al. 2021; Meade et al. 2015). Conversely, unexpected inverse associations for alcohol use in adjusted models likely reflect confounding, selection or survivor biases, variations in dose, frequency, timing, interactions with ART or comorbidities, or measurement error in self-reported use. Prior literature underscores that the cognitive effects of substances vary by class, recency, dose, route, and co-occurring psychiatric disorders; stimulants (e.g., cocaine, methamphetamine) show the clearest adverse associations with cognition in PWH (Litvin et al. 2021; Meade et al. 2015). Our results highlight the need for granular substance-use measurement and cautious interpretation of cross-sectional associations.

### Psychosocial and functional correlates

Persistently lower GAF scores among participants with HAND reinforce the clinical significance of HAND for everyday functioning and psychiatric morbidity. HAND often co-occurs with mood, anxiety, and substance-use disorders and may amplify disability and treatment nonadherence (Applebaum et al. 2009; Hu et al. 2024). The association of better psychosocial functioning (higher GAF) with reduced HAND risk highlights the critical role of mental health in neurocognitive outcomes among PWH (Fazeli et al. 2014; Treisman and Soudry 2016). The higher MCS scores observed in the HAND group are counterintuitive and may reflect a disconnection between subjective mental health reporting and objective functioning (e.g., self-report bias, somatic attribution, differing constructs captured by MCS versus clinician-rated GAF). Similar divergences between subjective and objective measures have been reported in HIV and psychiatric populations, reinforcing the need for integrated assessment approach combing clinician-rating, self-report, and objective neuropsychological testing (Hu et al. 2024; Shahriar et al. 2003). Additionally, baseline obesity was negatively associated with HAND. The protective role of obesity contrasts with literature linking metabolic syndrome to cognitive impairment (Guaraldi et al. 2011; Moran et al. 2013), however, one study found that central obesity measured by waist circumference, rather than BMI alone, increased the risk of neurocognitive impairment (NCI), and greater body mass may be protective if the deleterious effect of central obesity is accounted for (McCutchan et al. 2012).

### Longitudinal patterns and clinical implications for psychiatric practice

Two notable longitudinal patterns emerged. First, hematologic indices (hemoglobin and hematocrit) increased across the cohort, potentially reflecting improved medical management or recovery from reversible anemia; however, individuals with HAND remained relatively lower at follow-up, suggesting persistent biological vulnerability or incomplete recovery. Second, although psychological distress (GSI) declined over time, functional impairment (GAF) remained comparatively low among those with HAND. This dissociation indicates that reductions in subjective distress do not necessarily translate into improvements in functional capacity, highlighting the multidimensional nature of neurocognitive impairment.

These findings have important implications for both research and clinical care. From a research perspective, they underscore the need for longitudinal studies that simultaneously assess biological, psychological, and functional domains to better understand recovery trajectories in HAND. Clinically, the persistence of functional impairment despite improvements in distress supports the need for integrated care approaches that extend beyond symptom reduction. In particular, the observed associations between hematologic measures and neurocognitive status suggest that routine clinical indicators may serve as accessible signals for further cognitive evaluation, while the relationship with substance use highlights the importance of integrating addiction and psychiatric services. Together, these findings support a multidisciplinary care model that incorporates cognitive screening, functional rehabilitation (e.g., occupational therapy, cognitive remediation), and coordinated management across medical, psychiatric, and social domains to address the complex needs of individuals with HAND.

### Plausible biological mechanisms

The observed pattern, anemia/low red blood cell indices and platelet activation to HAND, reflects biologically plausible and convergent mechanisms. Anemia can reduce cerebral oxygenation and alter iron homeostasis, both critical for neuronal metabolism and neurotransmission (Kallianpur et al. 2016). Platelet activation and platelet–monocyte aggregates can compromise the blood–brain barrier and drive neuroinflammation by facilitating monocyte transmigration and microglial activation (Mesquita et al. 2018; Singh et al. 2014). Substance use, especially stimulants may exacerbate neuroinflammation, oxidative stress, and dopaminergic dysfunction, compounding HIV-related neural injury (Litvin et al. 2021). Psychosocial stress and psychiatric comorbidity may further increase systemic inflammation and allostatic load, thereby potentiating cognitive vulnerability (Hu et al. 2024). Together these pathways support a biopsychosocial model in which peripheral hematologic and inflammatory processes, substance-related neurotoxicity, and psychosocial factors interact to shape cognitive trajectories.

### Limitations

Several limitations should be considered when interpreting these findings. First, the data are archival and were collected approximately 9–14 years ago. During this period, substantial advances in ART, as well as changes in clinical management, diagnostic practices, and the epidemiology of PWH, have occurred. Consequently, the predictors and clinical presentation of HAND may have evolved, potentially limiting the generalizability of these results to contemporary populations. Furthermore, HAND classification relied on the IHDS, a screening tool rather than a diagnostic gold standard. The IHDS has limited sensitivity and specificity, particularly for milder forms of impairment, and does not substitute for comprehensive neuropsychological assessment. In addition, the study did not apply IHDS cutoff scores to distinguish HAND subtypes (e.g., ANI vs. MND), precluding subtype-specific analyses. Although HAD is now rare in the modern treatment era, the lack of detailed phenotyping limits clinical interpretability. Thus, findings should be interpreted as reflecting screening-level neurocognitive risk rather than definitive diagnoses.

Second, the study sample consisted exclusively of PWH who use substances. Because substance use can independently affect neurocognitive functioning and may interact with HIV-related factors, the identified predictors of HAND may not generalize to PWH who do not use substances. In addition, substance use was self-reported and assessed only over the past 30 days and past year, which may introduce recall and social desirability bias while limiting temporal resolution. The dichotomous operationalization (use vs. no use) further obscures variation in frequency, quantity, and severity. The absence of formal diagnoses of substance use disorders (SUDs) or measures of SUD severity restricts differentiation between occasional and clinically significant use. Moreover, substance use data were incomplete across categories (e.g., limited assessment of methamphetamine and non-heroin opioids), and smoking was measured without detailed exposure history (e.g., pack–years). Third, several measurement limitations warrant attention. Neurocognitive impairment prior to HIV infection, including that related to earlier substance use, could not be accounted for and may confound observed associations. Psychiatric comorbidities were assessed using global indices (e.g., GSI, MCS) rather than specific diagnostic categories, limiting the ability to evaluate disorder-specific effects such as major depressive disorder. Relatedly, SUDs as psychiatric conditions were not directly characterized. Furthermore, unmeasured confounding remains possible. The dataset lacked detailed information on ART regimens, nadir CD4 count, viral reservoir or cerebrospinal fluid (CSF) markers, inflammatory biomarkers (e.g., cytokines), iron indices, and precise substance-use timing and dose—all of which may influence neurocognitive outcomes. Incorporation of objective measures (e.g., toxicology data) would strengthen future analyses. Fourth, functional status was assessed using the GAF, which relies on clinician judgment and combines symptom severity with functional impairment, introducing subjectivity. This limitation is notable given the removal of GAF from DSM–5 due to concerns regarding its psychometric properties. The divergence observed between GAF and MCS may reflect differences between clinician-rated functioning and patient-reported mental health–related quality of life. Additionally, inter-rater reliability of GAF was not formally assessed. Future studies should incorporate standardized, multidimensional measures such as the World Health Organization Disability Assessment Schedule 2.0 (WHODAS 2.0). More broadly, reliance on self-report psychiatric measures (MCS, GSI) and the absence of a comprehensive neuropsychological battery (or adjustment for premorbid cognitive reserve) limit diagnostic precision. Fifth, some statistically significant findings (e.g., hemoglobin, hematocrit, platelet count) reflected small differences within normal clinical ranges, suggesting limited clinical significance. In particular, platelet count represents an indirect marker, and the absence of measures of platelet activation further constrains interpretation. Sixth, the analytic approach also has limitations. While linear mixed models capture average group-level change, they may not detect heterogeneous individual trajectories (e.g., subgroups with decline, stability, or improvement). More flexible approaches, such as growth mixture or random-slope models, could better characterize individual variability. Finally, generalizability may be limited by clinic- and region-specific sampling, and findings may differ across settings, particularly in low-resource environments. Future research using more recent cohorts, more detailed substance use and psychiatric assessments, and comprehensive neurocognitive evaluations is needed to confirm and extend these findings.

#### Conclusions and future directions

This RCT-based analysis indicates that HAND remains highly prevalent among people with HIV in the United States, with persistent sex and racial disparities. Individuals with HAND were more likely to be older, have lower educational attainment, be unemployed, and identify as African American, and they exhibited lower hemoglobin and hematocrit levels. In contrast, obesity and better psychosocial functioning were associated with lower likelihood of HAND, whereas higher platelet counts were associated with greater likelihood, although these effects were small and of limited clinical significance. Multivariable analyses further demonstrated that obesity, severe immunosuppression (CD4 < 200 cells/µL), hematologic indices, and functional status were significantly associated with neurocognitive performance. Logistic models indicated that alcohol use, viral suppression, higher hemoglobin and hematocrit levels, and better functioning were associated with reduced odds of HAND, while higher platelet count was associated with increased odds. Longitudinally, the cohort showed improvements in hematologic and psychosocial measures; however, individuals with HAND consistently exhibited poorer hematologic profiles and functional outcomes, suggesting incomplete recovery despite overall health gains. These findings highlight the complex interplay among hematologic, behavioral, and psychosocial factors in shaping neurocognitive outcomes. While the observed associations suggest potential clinical correlates, causal inferences cannot be made. Future research should incorporate comprehensive neuropsychological assessments, multimodal biomarkers, and more granular measures of substance use and psychiatric comorbidity. Longitudinal designs capable of identifying heterogeneous cognitive trajectories are needed, as are studies evaluating integrated care models that address medical, behavioral, and functional domains to improve outcomes for people with HIV at risk for HAND.

## Data Availability

The data that support the findings of this study are openly available in https://datashare.nida.nih.gov/study/nida-ctn-0049.
